# Vitamin D supplementation and serum heat shock protein 60 levels in patients with coronary heart disease: a randomized clinical trial

**DOI:** 10.1186/s12986-018-0292-9

**Published:** 2018-08-06

**Authors:** Leila Sadat Bahrami, Seyed Hashem Sezavar Seyedi Jandaghi, Leila Janani, Mina Pahlavan, Seyed Mostafa Arabi, Homa Sadeghi, Mohammadreza Vafa

**Affiliations:** 1 Department of Nutrition, School of Public Health, Iran University of Medical Sciences, Tehran, Iran; 2 Rasool-e Akram Hospital, Iran University of Medical Sciences, Tehran, Iran; 3 Department of Biostatistics, School of Public Health, Iran University of Medical Sciences, Tehran, Iran; 4 Department of Medical Surgical Nursing, Nursing and Midwifery School, Iran University of Medical Sciences, Tehran, Iran; 5 Department of Nutrition, Metabolic Syndrome Research Center, Mashhad University of Medical Sciences, Mashhad, Iran; 6 Institute of Public Health and Clinical Nutrition, University of Eastern Finland, Kuopio, Finland

**Keywords:** Vitamin D supplementation, Coronary heart disease, Heat shock protein 60

## Abstract

**Background:**

The aim in this study was to investigate the effect of vitamin D (25(OH)D_3_) supplementation on heat shock protein 60 (HSP 60) and other inflammatory markers (IL-17, TNF-α, PAB) in patients with coronary heart disease (CHD).

**Methods:**

In this double-blind, randomized clinical trial, we recruited 80 male and female patients aged 30–60 with CHD and 25(OH)D_3_ serum levels < 30 ng/ml from Rasool-e-Akram Hospital in Tehran, Iran. Serum levels of HSP 60 as primary outcome, and 25(OH)D3, IL-17, TNF-α, PAB, lipid profiles and parathyroid hormone (PTH) as secondary outcomes were measured at baseline and post-intervention. We randomly assigned eligible participants to a placebo group (*N* = 40) or an intervention group (N = 40) (50,000 IU/wk. vitamin D supplement) for eight weeks.

**Results:**

The results demonstrated that vitamin D supplementation resulted in a significant increase in 25(OH) D_3_ serum levels in the intervention group compared to the placebo group (46.86 vs. 7.28 ng/ml). PTH levels decreased in the intervention group compared to the placebo group (− 19.81 vs. 2.92 pg/ml) after eight weeks of supplementation. Furthermore, we observed a significant change in waist circumference (− 0.97 vs. -0.26 cm), fat percentage (−.13 vs. 0.1%), systolic blood pressure (− 3.85 vs. -2.11 mmHg) and diastolic blood presure (− 4 vs. -1.86 mmHg) in the vitamin D group compared to the placebo group (all *P* values < 0.05). Other variables did not significantly change after the intervention.

**Conclusion:**

Based on our findings, weekly vitamin D supplementation of 50,000 IU for eight weeks in patients with CHD resulted in decreased systolic and diastolic blood pressure, waist circumference and fat percentage. No significant effect on HSP 60, inflammatory markers or lipid profiles was observed.

**Trial registration:**

IRCT, IRCT201612122365N14. Registered 12 December 2016.

## Background

Cardiovascular disease (CVD) is one of the main causes of mortality and morbidity worldwide. Between 2011 and 2012, the direct and indirect costs of CVD and stroke were $316.6 billion in the US [1]. Coronary heart disease (CHD) accounts for the highest proportion of health care costs in the US, with more than 1 million hospitalizations per year [2]. In the Eastern Mediterranean region, 1.4 million deaths due to CVD occurred in 2015. In addition, disability adjusted life years (DALY) rates for CVD are higher than the global average in the Eastern Mediterranean region [3]. Atherosclerosis is the major cause of CVD. Even though the actual mechanism involved in the initiation of atherosclerosis remains unknown, there is a close correlation between inflammation and atherosclerosis both in the very early stages of atherosclerosis and in its progression [4]. The inflammatory cascade can be initiated by environmental or cardiovascular risk factors. Some cardiovascular risk factors are hemodynamic stress, sustained hyperlipidemia, homocysteinemia, smoking, inflammatory mediators and infection [4, 5]. Consequently, the inflammatory procedure may overlap plaque formation, endothelial injury and rupture [4].

Atherosclerotic lesions have been reported to increase the levels of growth factors and proinflammatory cytokines, such as HSPs [6]. HSPs are recognized according to their molecular weights as 110, 90, 70, 60, 40 kDa and lower molecular weights [7]. Under normal circumstances, HSPs play the role of molecular chaperones [8]. However, their production increases in stressful conditions, such as CVD, hypertension, systemic lupus erythematosus and dementia [9]. Classic atherosclerosis risk factors lead to the expression of endothelial HSP 60s and adhesion molecules. Because T cells respond to the HSP 60s from the beginning of atherogenesis, the presence of HSP 60 can be considered a ‘danger signal’ for both cellular and humoral defense response [10]. Moreover, some inflammatory markers, such as IL-17 [11] and TNF-α [12], participate in the pathogenesis of atherosclerosis.

Vitamin D is one the most common nutrients in studies on cardiovascular health. In addition to the pivotal role of vitamin D in muscloskeletal health via the metabolism of calcium, this vitamin may affect cardiovascular health via anti-inflammatory activity [4]. It has been estimated that approximately 50% of the world’s population has a vitamin D deficiency [13], and it has been suggested that this deficiency may increase the incidence of CVD worldwide [14, 15]. Vitamin D receptors are ubiquitous in all cells involved in atherosclerosis, such as vascular endothelial and immune cells [16].

It is thought that vitamin D prevents atherosclerosis through different mechanisms, such as the stimulation of nitric oxide bioavailability, the inhibition of smooth muscle proliferation in the endothelium and vascular calcification [17]. However, according to our knowledge, no study has been conducted on the effect of HSP 60. Therefore, the purpose of this study was to evaluate the effect of vitamin D supplementation on serum HSP 60 levels in patients with CHD.

## Methods

### Study design, participants and sample size

This randomized, placebo-controlled, double-blind trial was conducted at Rasool-e-Akram Hospital, Tehran, Iran from December 2016 to March 2017 during the autumn/winter months. The inclusion criteria were average age of 30–60 and a diagnosis CHD by a cardiologist. The exclusion criteria were BMI > 35 kg/m^2^; a 25(OH)D3 serum level > 30 ng/ml; any diseases such as cancer, myocardial infarction (MI), diabetes, liver or kidney disorders; the intake of any medication outside the treatment protocol; the intake of any nutritional supplement (oral and/or intravenous) in the previous four months; the intake of any herbal remedy; the routine consumption of vitamin D-fortified foods; pregnancy or lactation; smoking; and alcohol consumption. Our study population consisted of 40 participants in each group. We calculated the sample size by using G * Power 3 software. Cohen’s effect size was 0.7 for mean difference in the standard deviations. Among 110 enrollments, 80 males and females were eligible to participate the study. Eighty participants were randomly assigned in either placebo or vitamin D group (40 subjects in each group) after signing the consent form. The primary outcome of our study was HSP 60, and the secondary outcomes were Interleukin17 (IL17), Pro-oxidant-antioxidant Balance (PAB), TNF-alpha, low density lipoprotein (LDL) and high-density lipoprotein (HDL) cholesterol, anthropometric measurements (weight, Height, BMI, waist circumference, fat percentage), systolic and diastolic blood pressure. Prescreening of coronary heart disease was done by cardiologist in the first visit, and other inclusion and exclusion criteria were evaluated by our nutritionist. The study was approved by Ethical Committee of Iran University of Medical Sciences (Reference Number 28891), and was registered at the Iranian Registry of Clinical Trials (http://www.irct.ir) with the identification Number of IRCT201612122365N14.

### Randomization and intervention

We applied the block randomization method with quadruple blocks for randomized sampling. According to the sample size, 20 blocks were generated using (http://www.sealedenvelope.com) website. We filled similar boxes with vitamin D or placebo, and printed distinct random number on each box. For the sake of confidentiality of subjects’ information and blindness of the study, an outsider staff handed the boxes to the subjects according to the number of randomization. We followed up the subjects’ compliance by weekly phone calls.

Participants of the intervention group, received 50,000 IU cholecalciferol, and other 40 subjects of the placebo group took matching placebo (Zahravi pharm. Co; Tehran, Iran) for 8 weeks. Participants were advised to take their supplements once a week preferably with their lunch. Consumption of 50,000 IU vitamin D per week was considered to be safe since the upper limit is 10,000 IU/d [18].

### Anthropometric assessments and blood pressure

At the baseline and after the 8-week intervention period, anthropometric characteristics and blood pressure were measured with the same measurement tools and staff. Height was measured using a wall-mounted stadiometer with the nearest 0.1 cm. Weight and fat percentage were determined with a digital scale to the nearest 0.1 kg (Beurer, GmBH, Ulm, Germany). BMI was calculated as weight divided by the square of height (kg/m2). Waist circumference (WC) was measured at the approximate midpoint between the lower border of the last palpable rib and iliac crest in a standing position at the point of minimal waist via non-elastic meter to the nearest 0.1 cm. Blood pressure was measured 3 times at 5-min intervals in the seated position with both feet flat on the floor, after a 10-min rest, using a digital sphygmomanometer (Omron M3, Kyoto, Japan).

### Biomarkers analysis

Blood samples were collected at the baseline and after 8th-week intervention. Sera were separated from blood samples by 3000 g centrifugation in the room temperature and were kept in − 80 °C until analysis. Lipid profiles were measured with enzyme kits (Farasamed Co., Tehran, Iran) by an auto-analyzer (BT 1500). Serum level of 25-hydroxy vitamin D_3_ (25-(OH) D_3_) was evaluated by human ELISA kit (EUROIMMUN, Germany, cat.NO: EQ. 6411–9601). We categorized vitamin D status according to the following reference range: serum 25(OH) D_3_ levels < 10(ng/mL) as severe deficiency, 10–20(ng/mL) as deficiency, 20–30(ng/mL) as insufficiency, and > 30(ng/mL) as sufficiency [19]. Furthermore, the human ELISA kits of HSP 60 (ZellBio GmbH, Germany, cat.NO: ZB-11774S-H9648), IL-17 (ZellBio GmbH, Germany, cat.NO: ZB-10142S-H9648), TNF-α (IBL, Hamburg, Germany, cat.NO: BE55181), and PTH (EUROIMMUN, Germany, cat.NO: EQ. 6421–9601) were used to evaluate biomarkers. Serum concentration of PAB was measured based on the previous study protocol [20].

### Dietary intake, physical activity and sun exposure

We evaluated sun exposure, physical activity, and dietary status both at baseline and post-intervention. We asked the subjects about the duration of sun exposure in minutes per hours of a routine day in the recent week. Accordingly, the duration of sun exposure was categorized as 0–10 min; 10 min–1 h; 1–2 h; and more than 2 h. We asked them about the exact time of exposure as well: 7 am–10 am, 10 am–3 pm, and 3 pm–5 pm. For autumn and winter days, we only considered the duration of sun exposure between 10 am and 3 pm. Furthermore, the participants were asked which parts of their body were exposed to sunlight and whether they used sunscreen or not. Physical activity (Met-Min/week) level was measured by the standardized short-form of the International Physical Activity Questionnaire (IPAQ) in Persian [21]. We evaluated Dietary intakes by 2-day food recall forms (1 usual day and 1 holiday) and the Nutritionist IV software program (First Databank Inc., Hearst Corp., San Bruno, CA, USA).

### Statistical analysis

We tested normal distribution via Kolmogorov-Smirnov with *P* value of 0.05. We tested between-group differences by independent sample t-test and Mann-Whitney U test as the parametric and nonparametric tests respectively. Between-groups variance was assessed by analysis of covariance (ANCOVA) for weight, BMI, energy, dietary calcium, vitamin D intake, sun exposure, physical activity, and baseline values as covariates. Besides, paired t test and nonparametric Wilcoxon signed-rank test were applied for assessing within-group differences (TNF-α, PAB, 25(OH) D3, LDL, HDL, TC, TG, PTH, Energy, Vitamin D, Calcium, BMI, WC, Weight, Fat percentage, Systolic blood pressure, Diastolic blood pressure). For comparing the difference between observed and expected frequency we ran Chi-square test. All statistically significant analyses were run in SPSS (ver. 24; SPSS Inc.، Chicago، IL، USA). A *P* value less than 0.05 was considered significant.

## Results

### Participants’ enrolment

**Fig. 1:**
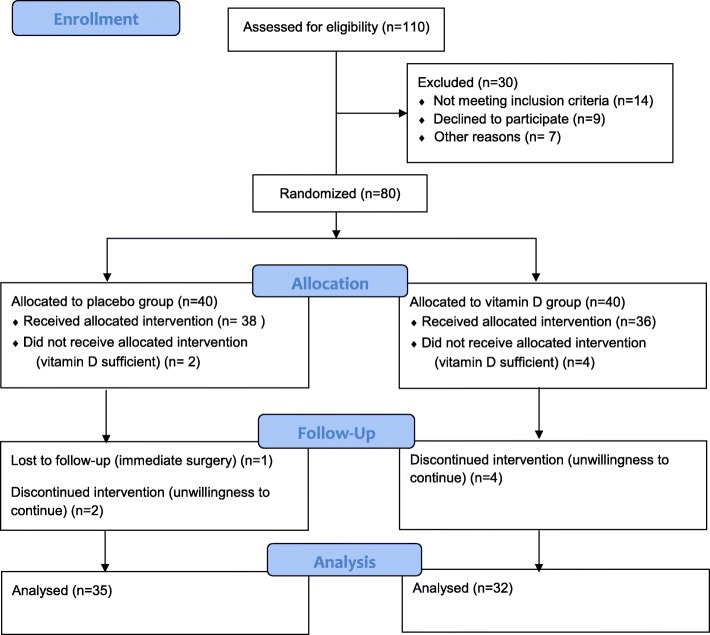
Flow chart. Of the 110 subjects assessed for eligibility 67 completed the study stages

Among 110 enrolled participants, 80 subjects were eligible for inclusion, and 67 patients completed 8 weeks follow up. We didn’t receive any reports of adverse side effects or vitamin D toxicity symptoms during the trial. [Fig. 1].

### Demographic characteristics

The mean age of the participants was 56 years. Approximately, most of the subjects were males (71.9% in vitamin D and 77.1% in placebo group), and most of them were employed (59.4% in vitamin D and 57.1% in placebo group). At baseline, there were no differences between placebo and intervention groups (*p* > 0.05). [Table 1].

**Table 1:** Baseline Characteristics of the Study Participants

	Placebo (35 patient)	Vitamin D (32 patient)	*P* value
Age^a^	56.00 (46, 60)	56.00 (53, 59.75)	0.717^1^
Weight (kg)	82.12 (10.07)	79.01 (11.17)	0.233^2^
Height (cm)	168.8 (8.3)	167.8 (7.9)	0.633^2^
BMI (kg/m^2^)	28.4 (3.37)	27.05 (3.68)	0.301^2^
WC (cm)	94.17 (10.29)	92.43 (12.48)	0.536^2^
Fat percentage (%)	32.65 (4.57)	33.97 (6.45)	0.332^2^
Systolic blood pressure (mmHg)	126.51 (12.70)	129.00 (15.82)	0.479^2^
Diastolic blood pressure (mmHg)	80.14 (8.7)	79.93 (10.61)	0.931^2^
Gender			
Male, Frequency (%)	27 (77.1)	23 (71.9)	0.621^3^
Female, Frequency (%)	8 (22.9)	9 (28.1)	
Job			
Employed, Frequency (%)	20 (57.10)	19 (59.40)	0.774^3^
Unemployed, Frequency (%)	5 (14.30)	6 (18.70)	
Retired, Frequency (%)	10 (28.60)	7 (21.90)	

^a^For descriptive statistics, we used Man-Whitney test for Between groups comparisons (Median (IQR)). ^1^Statistical significance is based on Man-Whitney test, ^2^Statistical significances are based on independent t-test (Mean (SD)), ^3^Statistical significances are based on Chi-square test. BMI, Body Mass Index; WC, Waist Circumference

Vitamin D supplementation had no effect on HSP 60 serum concentration (*p* > 0.05). IL-17, TNF-α, PAB and Lipid profiles did not change significantly within and between groups. There was a significant decrease in PTH level (− 19.81 vs. 2.92 pg/ml) (*p* = 0.003), and a dramatic increase in 25(OH) D_3_ serum levels in intervention group and the modest increase in placebo group (46.86 vs. 7.28 ng/ml) (*p* <  0.001 vs 0.016). Dietary factors including energy, vitamin D, and calcium did not significantly differ between and within 2 groups at the baseline vs after 8 weeks intervention (*p* > 0.05). Anthropometric characteristics and blood pressure are represented in Table 2. BMI and weight did not change significantly after intervention (p > 0.05). Waist circumference (− 0.97 vs. -0.26 cm) (*p* = 0.005), systolic (− 3.85 vs. -2.11 mmHg) (*p* = 0.009) and diastolic blood pressure (− 4 vs. -1.86 mmHg) (*p* = 0.010), and fat percentage (− 1.13 vs. 0.1%) (*p* = 0.029), decreased significantly in vitamin D group after intervention. Whereas, diastolic blood pressure of placebo group attenuated after the intervention [Table 2].

**Table 2:** Biochemistry Tests Dietary Intake, Anthropometric Characteristics and Blood Pressure at the Baseline and After Intervention

	Placebo (35 patient)	Vitamin D (32 patient)	*P* value^2^
HSP 60^a^ (ng/l)			
Baseline	599.40 (478.80, 1089.00)	603.15 (498.12, 1416.50)	0.880
Endpoint	765.40 (566.80, 1450.00)	649.20 (453.57, 1138.25)	0.142
*P* value^1^	0.083	0.104	
IL17^a^ (ng/l)			
Baseline	77.70 (69.50, 183.50)	86.55 (76.27, 122.52)	0.251
Endpoint	82.00 (70.70, 161.20)	77.00 (69.30, 130.80)	0.585
*P* value^1^	0.187	0.183	
TNFα (pg/ml)			
Baseline	2.18 (01.86)	2.10 (1.62)	0.894
Endpoint	2.79 (2.68)	3.04 (3.48)	0.734
*P* value^1^	0.095	0.111	
PAB (HK)			
Baseline	148.02 (69.07)	168.56 (71.39)	0.236
Endpoint	149.89 (65.54)	166.50 (62.29)	0.293
*P* value^1^	0.820	0.866	
25(OH) D3 (ng/ml)			
Baseline	17.26 (6.95)	16.62 (7.46)	0.717
Endpoint	27.54 (25.88)	63.48 (25.86)	< 0.001
*P* value^1^	0.016	< 0.001	
LDL (mg/dl)			
Baseline	81.48 (22.17)	89.37 (26.78)	0.192
Endpoint	80.34 (29.41)	84.28 (29.49)	0.586
*P* value^1^	0.722	0.162	
HDL (mg/dl)			
Baseline	37.68 (7.42)	35.81 (7.50)	0.308
Endpoint	35.68 (9.94)	34.96 (7.54)	0.743
*P* value^1^	0.163	0.459	
TC (mg/dl)			
Baseline	124.05 (29.89)	134.37 (33.05)	0.184
Endpoint	124.77 (37.01)	128.09(38.08)	0.719
*P* value^1^	0.858	0.153	
TG (mg/dl)			
Baseline	136.48 (75.73)	154.50 (89.96)	0.377
Endpoint	153.02 (80.01)	172.37 (98.19)	0.382
*P* value^1^	0.160	0.124	
PTH (pg/ml)			
Baseline	37.80 (15.08)	43.35 (23.31)	0.258
Endpoint	40.72 (22.61)	23.54 (17.10)	0.248
*P* value^1^	0.306	0.003	
Energy (cal)			
Baseline	1733.28 (297.96)	1859.48 (327.65)	0.103
Endpoint	1776.27 (292.26)	1889.46 (298.51)	0.122
*P* value^1^	0.403	0.617	
Vitamin D^a^ (ug)			
Baseline	1.34 (0.08, 2.64)	0.94 (0.36, 2.14)	0.660
Endpoint	1.56 (0.96, 2.61)	1.39 (0.66, 2.41)	0.514
*P* value^1^	0.647	0.121	
Calcium (mg)			
Baseline	720.13 (250.60)	723.90 (217.96)	0.948
Endpoint	807.77 (243.86)	785.90 (244.23)	0.715
*P* value^1^	0.108	0.266	
BMI (kg/m^2^)			
Baseline	28.4 (3.37)	27.05 (3.68)	0.301
Endpoint	28.31 (3.34)	27.37 (3.64)	0.276
*P* value^1^	0.475	0.423	
WC (cm)			
Baseline	94.17 (10.29)	92.43 (12.48)	0.536
Endpoint	93.91 (10.38)	91.46 (12.62)	0.388
*P* value^1^	0.324	0.005	
Weight (kg)			
Baseline	82.12 (10.07)	79.01 (11.17)	0.233
Endpoint	81.85 (9.69)	78.27 (70.79)	0.158
*P* value^1^	0.319	0.103	
Fat percentage (%)			
Baseline	32.65 (4.57)	33.97 (6.45)	0.332
Endpoint	32.75 (4.81)	32.84 (5.79)	0.943
*P* value^1^	0.729	0.029	
Systolic blood pressure (mmHg)			
Baseline	126.51 (12.70)	129.00 (15.82)	0.479
Endpoint	124.4 (8.29)	125.15 (14.16)	0.796
*P* value^1^	0.146	0.009	
Diastolic blood pressure (mmHg)			
Baseline	80.14 (8.7)	79.93 (10.61)	0.931
Endpoint	78.28 (7.46)	75.93 (8.74)	0.240
*P* value^1^	0.003	0.010	

^a^ For descriptive statistics, Man-Whitney test was used for between-groups comparisons (Median (IQR)). Wilcoxon test was used for within-groups comparisons (Mean (SD)).^1^Within-group *P* values based on paired t-test, ^2^ Between group *P* values based on t-test. ^3^ Post-intervention between-group P value based on ANCOVA with baseline value of each variable and BMI as covariates. HSP 60, IL17, Interleukin 17; *TNFα* Tumor necrosis factor α, *PAB* Pro oxidant Anti-oxidant Balance, *LDL* Low density lipoprotein, *HDL* High Density Lipoprotein, *TC* Total Cholesterol, *TG* Triglyceride, *PTH* Parathyroid Hormone, *BMI* Body Mass Index, *WC* Waist Circumference

Hours of exposure to sunlight and physical activity did not differ significantly between groups at the baseline and post intervention. (p > 0.05) [Table 3].

**Table 3:** Sun Exposure and Physical Activity at Baseline and After Intervention

	Placebo (35 patient)	Vitamin D (32 patient)	*P* value^1^
Sun exposure			
Baseline			
less than 1 h/day	19 (54.30)	20 (62.50)	0.496
more than 1 h/day	16 (45.70)	12 (37.50)	
Endpoint			
less than 1 h/day	19 (54.30)	18 (56.30)	0.872
more than 1 h/day	16 (45.70)	14 (43.80)	
Physical Activity (MET-minute/Week)			
Baseline			
Light	22 (62.90)	21 (65.60)	0.813
moderate & vigorous	13 (37.10)	11 (34.40)	
Endpoint			
Light	19 (54.30)	16 (50)	0.726
moderate & vigorous	16 (45.70)	16 (50)	

^1^Between-group *P* values based on Chi- square test

## Discussion

According to the results of our study, vitamin D supplementation increased serum vitamin D levels comparing placebo group. However, HSP-60 did not change significantly after intervention (*p* = 0.08). Oral intake of 50,000 IU vitamin D once a week for 8 weeks a standard protocol for treatment of severe and moderate vitamin D deficiency, and no adverse effects have been reported in human studies for this recommendation [19, 22]. Various interventional and cross-sectional studies have been carried out in this field and have yielded concordant results from our study [23–25].

### HSP 60

There was no significant difference between HSP 60 levels in placebo group and vitamin D group before and after intervention. Oxidative stress, infection, heat, and immune diseases may stimulate body production of HSP 60, and conclusively increase inflammatory complications [26]. Various studies have suggested that vitamin D may reduce oxidative stress [27], which decreases HSP 60 production. However, the results of our research, represented controversial results about the association of vitamin D and HSP 60.

### Inflammatory markers

Our Findings indicated no significant differences in serum levels of inflammatory markers within and between groups. Various studies have been conducted on the effect of vitamin D supplementation on inflammation. In line with the results of our study, Witham and coworkers reported no differences in TNF-α, among MI patients (*N* = 74) treated with single dose of 100,000 IU vitamin D comparing placebo group [28]. Nonsignificant results may have been due to low bioavailable dose of vitamin D in each intervention episode. Similarly, Jorde and colleagues indicated that vitamin D supplementation didn’t have any effects on IL-17 and other inflammatory markers in patients with overweight and obesity [29]. In contrast, Schleithoff and coworkers compared the effect of 50 μg vitamin D plus 500 mg calcium versus placebo in a daily manner for 9 months, among 93 CHD patients. They found that TNF-α (inflammatory marker) decreased, and IL-10 (anti-inflammatory marker) increased significantly after vitamin D intervention [30]. The inconsistent results may be related to baseline vitamin D status/25(OH)D concentrations of study subjects, intervention dose, length of intervention, bioavailability of supplements, and added effect of calcium.

### PAB

In the current study, there was no significant association between vitamin D supplementation and PAB. Asemi and colleagues investigated the effect of 50,000 IU vitamin D comparing placebo on total Antioxidant Capacity (TAC) among 54 patients with Gestational diabetes mellitus (GDM) and vitamin D deficiency. No relationship was found between vitamin D supplementation and oxidative stress levels. These results may have been due to low baseline levels of oxidative stress [27]. In contrast, Anandabaskar and coworkers demonstrated that weekly supplementation of 60,000 IU vitamin D for 8 weeks among 70 males and females with type 2 diabetes may decrease malondialdehyde (MDA) and TAC levels [31].

### Lipid profiles

Vitamin D may affect lipid profiles by reducing intestinal absorption of lipids, endogenic lipid synthesis, stimulating lipolysis, and improving lipid metabolism [32]. Vitamin D may improve insulin activity that prevents metabolic syndrome [32]. Furthermore, vitamin D may inhibit bile acid synthesis which results in decreased cholesterol levels [32]. However, we did not find any significant results around improvement of lipid profiles. The results of our study are along with some other previous studies which failed to demonstrate any significant association between increased serum level of vitamin D and improvement of the lipid profiles [33–35]. Non-significant results may have been because of normal concentration of lipid profiles at the baseline. On the other side, Salekzamani and coworkers demonstrated that 50,000 IU/week vitamin D for 16 weeks resulted in descending Triglyceride (TG) levels in 80 subjects with vitamin D deficiency and metabolic syndrome [36].

### PTH

vitamin D deficiency leads to hyperactivity of parathyroid gland to compensate for lack of vitamin D, as the result of which PTH level increases [37]. In the current study, vitamin D supplementation represented a significant decrease in PTH level after vitamin D intervention. According to the results of previous studies, increased levels of parathyroid hormone may lead to the transfer of large amounts of calcium into cardiomyocyte and can lead to death of these cells and damage to the heart tissue. Also lead to contractions and tightness of vessels in the smooth muscle of vascular endothelium wall that can be effective in boosting blood pressure. Therefore, supplementation of vitamin D that can reduce parathyroid hormone levels may be effective in improving cardiovascular health [38].

### Anthropometric measurements, dietary intake, physical activity and sun exposure

The covariates of this study were weight, BMI, WC, body fat percentage, dietary intake of energy, vitamin D, calcium, physical activity, and sun exposure. WC and body fat percentage represented significant decrease in vitamin D group after the intervention. Mean systolic and diastolic blood pressure significantly decreased in vitamin D group after the intervention. On the other side, diastolic blood pressure was significantly lower within placebo group comparing baseline and endpoint.

### Blood pressure

Various observational studies have reported an inverse association between vitamin D levels and blood pressure [38–40]. The probable biological mechanisms for aforementioned studies may be related to the expression of vitamin D receptors on the surface of endothelial cells, smooth muscle cells, and myocytes [41–43]. Furthermore, vitamin D may improve endothelial function and decrease production of pro-inflammatory cytokines, renin-angiotensin system activity, and PTH level [38, 44].

### Strengths and limitations

We applied the safe and sufficient dose of vitamin D, which could replace serum level of 25 (OH) D3 correctly. Moreover, based on our information this study was conducted for the first time. We controlled the study for potential confounding factors as well. However, our study had some limitations such as short study period, using the ELISA method to measure 25(OH) D3, and enrolling the study subjects before measuring 25 (OH) D3 levels, because of which, 6 subjects were excluded since they were non-deficient.

## Conclusion

In conclusion, vitamin D supplementation only represented the significant effect on 25(OH) D_3_, PTH, WC and fat percentage in subjects with coronary heart disease. In short-term treatment, it is difficult to evaluate the efficacy of vitamin D supplementation. To confirm the beneficial effect of vitamin D on health outcomes, longitudinal clinical trials are required. We recommended future studies to assess the effect of vitamin D supplementation on another types of HSPs in CVD patients in a larger sample size and longer follow-up period. The results of this study can be used in future studies with similar theme of research, and health-related strategic planning for CVD in countries with similar socioeconomic, cultural, and vitamin D deficiency prevalence.

## Abbreviations

BMI: Body Mass Index
HDL: High Density Lipoprotein
HSP: Heat Shock Protein
IL: Interleukin
LDL: Low Density Lipoprotein
PAB: Pro oxidant Anti-oxidant Balance
PTH: Parathyroid Hormone
TC: Total cholesterol
TG: Triglyceride
TNF: Tumor Necrosis Factor
WC: Waist Circumference
